# Supplementary material for: A KAN-based hybrid deep neural networks for accurate identification of transcription factor binding sites
Source: PLoS One. 2025 May 7;20(5):e0322978. doi: 10.1371/journal.pone.0322978 (PMC12058130; doi:10.1371/journal.pone.0322978)
Supplement: Attachment — Submitted filename: Response__to_Reviewers-20250302_auresp_2.docx [file pone.0322978.s003.docx]

Reviewer #2: Although the authors have addressed some concerns, I am still doubtful about the generalizability of the model. I think the authors should perform cross-cell validation for the same.

**Answer:** Thank you for your meticulous review and valuable comments on our paper. We highly value your suggestion regarding the generalization ability of the model and have conducted further experimental verification based on your feedback. To further validate the robustness and generalisability of our model across diverse cell lines, we sourced data from three additional cell lines available on GEO: GSM1873040 (LNCaP), GSM2437758 (MCF - 7), and GSM2067524 (A549). Subsequently, we applied our model to these datasets, and the outcomes are presented in Table 5.

Table 5 Comparison with existing models across diverse cell lines.

| Datasets | Model | ROC AUC | Accuracy | F1-score | MCC |
| --- | --- | --- | --- | --- | --- |
| GSM1873040  （LNCaP） | DeepBind | 0.935 | 0.915 | 0.735 | 0.693 |
|  | DanQ | 0.948 | 0.923 | 0.753 | 0.722 |
|  | DeepD2V | 0.961 | 0.936 | 0.805 | 0.772 |
|  | DeepSEA | 0.957 | 0.934 | 0.813 | 0.773 |
|  | CBR-KAN | **0.964** | **0.941** | **0.821** | **0.792** |
| GSM2437758  (A549) | DeepBind | 0.968 | 0.936 | 0.851 | 0.812 |
|  | DanQ | 0.981 | 0.961 | 0.911 | 0.887 |
|  | DeepD2V | 0.990 | 0.977 | 0.947 | 0.932 |
|  | DeepSEA | 0.991 | 0.976 | 0.945 | 0.930 |
|  | CBR-KAN | **0.993** | **0.977** | **0.948** | **0.934** |
| GSM2067524  (MCF-7) | DeepBind | 0.976 | 0.928 | 0.879 | 0.829 |
|  | DanQ | 0.981 | 0.936 | 0.882 | 0.840 |
|  | DeepD2V | 0.992 | 0.961 | 0.927 | 0.902 |
|  | DeepSEA | 0.991 | 0.963 | 0.934 | 0.908 |
|  | CBR-KAN | **0.993** | **0.968** | **0.944** | **0.922** |

As shown in Table 5, the CBR-KAN model proposed in this study demonstrates significant superiority over existing models across all core metrics (ROC AUC, accuracy, F1-score, MCC) on three cell line datasets: LNCaP, A549, and MCF-7, validating its exceptional robustness and cross-cell-line generalizability. CBR-KAN achieves the highest ROC AUC values (LNCaP: 0.964 vs. 0.961; A549: 0.993 vs. 0.991; MCF-7: 0.993 vs. 0.992) and MCC scores (e.g., 0.922 vs. 0.902 in MCF-7), demonstrating its enhanced capability to capture complex genomic sequence features. The model exhibits particularly notable advantages in class-imbalanced scenarios, as evidenced by a 2.4% relative improvement in MCC compared to the second-best model on the LNCaP cell line dataset.

We believe that these supplementary experiments further demonstrate the broad applicability and stability of the CBR - KAN model across different cell lines. We have added this part to the paper and hope that these results can address your concerns about the generalization ability of the model.

Thank you again for your valuable comments.
